# The Impact of Spring Festival Travel on Epidemic Spreading in China

**DOI:** 10.3390/v15071527

**Published:** 2023-07-10

**Authors:** Hao-Chen Sun, Sen Pei, Lin Wang, Yuan-Yuan Sun, Xiao-Ke Xu

**Affiliations:** 1 College of Computer Science and Technology, Dalian University of Technology, Dalian 116024, China; sunhaochen@mail.dlut.edu.cn (H.-C.S.); syuan@dlut.edu.cn (Y.-Y.S.); 2 Department of Environmental Health Sciences, Mailman School of Public Health, Columbia University, New York, NY 10032, USA; 3 Department of Genetics, University of Cambridge, Cambridge CB2 3EH, UK; lw660@cam.ac.uk; 4 Computational Communication Research Center, Beijing Normal University, Zhuhai 519087, China; 5 School of Journalism and Communication, Beijing Normal University, Beijing 100875, China

**Keywords:** Spring Festival travel, epidemic spread, transportation network, metapopulation model, China

## Abstract

The large population movement during the Spring Festival travel in China can considerably accelerate the spread of epidemics, especially after the relaxation of strict control measures against COVID-19. This study aims to assess the impact of population migration in Spring Festival holiday on epidemic spread under different scenarios. Using inter-city population movement data, we construct the population flow network during the non-holiday time as well as the Spring Festival holiday. We build a large-scale metapopulation model to simulate the epidemic spread among 371 Chinese cities. We analyze the impact of Spring Festival travel on the peak timing and peak magnitude nationally and in each city. Assuming an *R*_0_ (basic reproduction number) of 15 and the initial conditions as the reported COVID-19 infections on 17 December 2022, model simulations indicate that the Spring Festival travel can substantially increase the national peak magnitude of infection. The infection peaks arrive at most cities 1–4 days earlier as compared to those of the non-holiday time. While peak infections in certain large cities, such as Beijing and Shanghai, are decreased due to the massive migration of people to smaller cities during the pre-Spring Festival period, peak infections increase significantly in small- or medium-sized cities. For a less transmissible disease (*R*_0_ = 5), infection peaks in large cities are delayed until after the Spring Festival. Small- or medium-sized cities may experience a larger infection due to the large-scale population migration from metropolitan areas. The increased disease burden may impose considerable strain on the healthcare systems in these resource-limited areas. For a less transmissible disease, particular attention needs to be paid to outbreaks in large cities when people resume work after holidays.

## 1. Introduction

Human mobility can shape the transmission of epidemics at different geographical scales [1,2,3,4,5,6]. Assessing how human movement impacts key epidemiological characteristics of an outbreak, such as peak timing [7,8,9] and peak magnitude [10,11] of infections, can improve the preparedness for new outbreaks and policy-making for controlling epidemic spread.

Mass vaccination can effectively reduce the incidence of severe illness caused by Omicron [12]. With a high vaccination rate of Chinese residents and the circulation of the less severe Omicron variants in many cities, China lifted its national prevention and control policies on 7 December 2022. After the relaxation of epidemic control, it is expected that the Spring Festival travel would likely return to the pre-pandemic level. The large-scale human movement during the Spring Festival travel can accelerate the spread of Omicron waves in a largely susceptible population. Understanding the impact of such massive population migration on the spread of Omicron and, more broadly, other infectious diseases can inform better resource planning and improved mitigation measures. To develop appropriate epidemic prevention measures for the Spring Festival travel population movement and virus transmission characteristics, which will help people to travel normally for the festival and resume work, production and school smoothly after the festival.

We use the pre-pandemic level inter-city mobility data to simulate population movement across 371 Chinese cities after relaxing epidemic control measures. We construct a large-scale metapopulation model using the inter-city traffic data. Using COVID-19 as a motivating example, we analyze the impact of the Spring Festival travel on epidemic spread at both national and city levels. Note that we do not aim to project the actual spread of Omicron across China; instead, our objective is to use mathematical models and simulations to study the impact of the Spring Festival travel on general epidemic spread. In addition, we also run model simulations under different *R*_0_ to highlight the impact of the Spring Festival travel on epidemics with various levels of disease transmissibility.

## 2. Materials and Methods

### 2.1. Data Collection

Using traffic flow data for epidemic simulation is an effective method [13]. This study uses the inter-city population flows among 371 cities across 34 provinces in 2018, which was provided by the Tencent location-based services. Daily numbers of travelers between each pair of 371 cities were recorded each day from 1 January 2018 to 31 December 2018. The mobility data in 2018 are assumed to be representative of the inter-city traffic after the relaxation of epidemic prevention measures. This dataset has supported several modeling studies on epidemic spread [14,15,16].

### 2.2. The City-Level Transmission Model

#### 2.2.1. Using Mobility Data

According to the official definition, the 15 days before the Spring Festival and the 25 days (including the Spring Festival) after the Spring Festival, i.e., 1 February to 12 March 2018, were considered as the Spring Festival travel season. Other major official Chinese holidays include New Year’s Day (30 December 2017–1 January 2018), Lantern Festival (2 March 2018), Tomb-sweeping Festival (5 April 2018–7 April 2018), Labor Day (29 April 2018–1 May 2018), Dragon Boat Festival (16 June 2018–18 June 2018), Mid-Autumn Festival (22 September 2018–24 September 2018), and National Day (1 October 2018–7 October 2018). This study considers the dates outside of these major official Chinese holidays as the non-holiday time.

We simulated the spread of epidemics during the period of non-holiday time (i.e., 20 November to 29 December 2018) versus during the Spring Festival travel season (i.e., 1 February to 12 March 2018).

#### 2.2.2. Model Framework

The partitioned model includes interactions between various categories of individuals (e.g., susceptible individuals, infectious individuals, cured individuals), which is central to many epidemiologic models. Simulation of disease transmission using mathematical models is an effective method for predicting epidemic development [17,18,19,20,21,22], such as ordinary differential equation (ODE) models [23,24] and partial differential equation (PDE) models [25,26]. This study focuses on models of infectious disease in the context of population mobility patterns, which requires consideration of not only individual shifts in infection status but also spatial changes in the population of cities. We developed a mathematical model to simulate the spatiotemporal dynamics of epidemic transmission among 371 Chinese cities [14]; the model takes into account the population movement of people between cities. The transmission model incorporates information on human movement within the following metapopulation structure [27,28,29]:

(1) $$\frac{dS_{i}}{dt}=-\frac{\beta S_{i}I_{i}^{r}}{N_{i}}-\frac{\mu {\beta S}_{i}I_{i}^{u}}{N_{i}}+\theta \sum_{j}\frac{M_{ij}S_{j}}{N_{j}-I_{j}^{r}}-\theta \sum_{j}\frac{M_{ji}S_{i}}{N_{i}-I_{i}^{r}}$$

(2) $$\frac{dE_{i}}{dt}=\frac{\beta S_{i}I_{i}^{r}}{N_{i}}+\frac{\mu {\beta S}_{i}I_{i}^{u}}{N_{i}}-\frac{E_{i}}{Z}+\theta \sum_{j}\frac{M_{ij}E_{j}}{N_{j}-I_{j}^{r}}-\theta \sum_{j}\frac{M_{ji}E_{i}}{N_{i}-I_{i}^{r}}$$

(3) $$\frac{dI_{i}^{r}}{dt}=\alpha \frac{E_{i}}{Z}-\frac{I_{i}^{r}}{D}$$

(4) $$\frac{dI_{i}^{u}}{dt}=\left(1-\alpha \right)\frac{E_{i}}{Z}-\frac{I_{i}^{u}}{D}+\theta \sum_{j}\frac{M_{ij}I_{j}^{u}}{N_{j}-I_{j}^{r}}-\theta \sum_{j}\frac{M_{ji}I_{i}^{u}}{N_{i}-I_{i}^{r}}$$

(5) $$\frac{dN_{i}}{dt}=\;\theta \sum_{j}M_{ij}-\theta \sum_{j}M_{ji}$$

where $S_{i}$, $E_{i}$, $I_{i}^{r}$, $I_{i}^{u}$, and $N_{i}$ are the susceptible, exposed, documented infected, undocumented infected, and total population in city *i*. We define patients with symptoms severe enough to be confirmed as documented infected individuals, whereas other infections are defined as undocumented infected individuals. We define a transmission rate *β* for documented infected individuals. The transmission rate for undocumented individuals is reduced by multiplying a factor μ. In addition, α is the fraction of documented infections, *Z* is the latency period, and *D* is the duration of infection. Spatial coupling within the model is represented by the daily number of people traveling from city *j* to city *i* ($M_{ij}$) and a multiplicative factor θ, which corrects for reporting bias of human movement. We assume that individuals in the $I_{i}^{r}$ group do not move between cities, though these individuals can move between cities during the latency period. In this study, each city is defined separately as a spatial system, and the elementary jump (population movement) of each spatial system is calculated based on empirical traffic flow data in which the actual population movement between each city system is included.

The basic reproduction number (*R*_0_) is an indicator of the infectiousness or transmissibility of infectious and parasitic agents; it is defined as the average number of secondary cases generated by a case in a fully susceptible population [30]. The *R*_0_ of an infectious disease event is usually reported as a single value or low–high range; if the value of *R*_0_ is >1, the outbreak continues, and if the value of *R*_0_ is <1, the outbreak will end [31]. The potential size of an outbreak or epidemic is usually based on the magnitude of the *R*_0_ value for that event [32].

The *R*_0_ value used in the manuscript was calculated by the researchers, not our own derivation. We used the epidemiological characteristics of Omicron in model simulations as the primary analysis. The basic reproductive number *R*_0_ was set to 15, the median value of *R*_0_ between Omicron BA.2 and BA.5 [33]. We assumed that $I_{i}^{r}$ and $I_{i}^{u}$ have the same infectivity, setting μ as 1. Considering possible double counting of travelers in the Tencent data, we set *θ* to 0.40. We further set the latency period as 2 days and the duration of infection as 5 days [33], the fraction of infections that develop severe symptoms α as 0.033 [34], and the transmission rate β as 3 computed using the equation $R_{0}=\;\alpha \beta D\;+(1-\alpha )\mu \beta D$ [14]. We used the sum of $E_{i}$*,* $I_{i}^{r}$, and $I_{i}^{u}$ to represent the total number of active infections in each city. To initiate the outbreak, we set the initial infections in each city as the confirmed COVID-19 cases as of 17 December 2022 (after the control relaxation).

This study considers only the virus transmission during the Spring Festival travel season and uses forty days of traffic flow data. It is difficult to reach steady states, and therefore we did not study the steady states of the model. Since this study focuses on the effect of Spring Festival travel on susceptible and infected individuals, the removal state individuals were not reflected in the formula. However, in order to satisfy the compartment conservation law of the model, the removal of infectious individuals was present in the actual simulation.

## 3. Results

We used model simulations to examine the impact of Spring Festival travel on the spatiotemporal transmission of epidemics. Specifically, we focused on two metrics that are crucial for policy-making and disease control: peak timing and peak magnitude for active infections. Peak timing is defined as the date with the largest number of active infections (the infection population (the sum of exposed population, documented infected population, undocumented infected population) as a proportion of the total population); peak magnitude represents the fraction of active infections at the peak among the total population.

**Figure 1:**
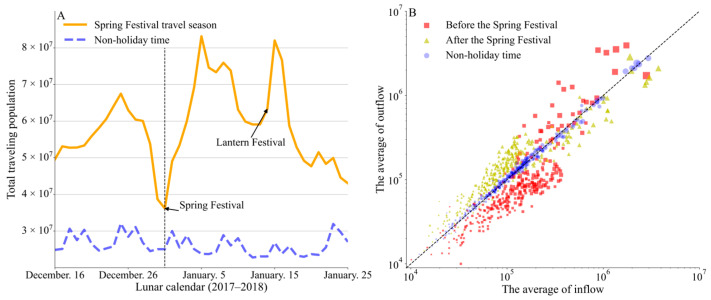
National traffic flow during the Spring Festival travel season and the non-holiday time in 2018. (**A**) The total traveling population during the Spring Festival holidays (Orange) and non-holidays (Blue). The dates on the x-axis correspond to the orange lines (Spring Festival travel season), and the blue line corresponds to the traffic flow from 20 November to 29 December 2018 without holidays. (**B**) The average outflow and inflow of each city before the Spring Festival and after the Spring Festival during the Spring Festival travel season, and during the non-holiday time.

As shown in Figure 1A, the traffic flows during the 2018 Spring Festival travel season were significantly greater than during the non-holiday time. The Spring Festival travel season began with an increase in nationwide traffic. Population flow gradually decreased before the Spring Festival and Lantern Festival, and the traveling population continued to grow after the two festivals when people resumed work. At the end of the Spring Festival travel season, the traveling population gradually decreased.

A clear asymmetric travel pattern emerged during the Spring Festival travel (Figure 1B). Before the Spring Festival (the period of returning home), people mainly traveled from large cities to small- or medium-sized cities; after the Spring Festival (the period of returning to work), the population traveled in reverse directions. In contrast, population outflow and inflow during the non-holiday time were almost symmetric.

**Figure 2:**
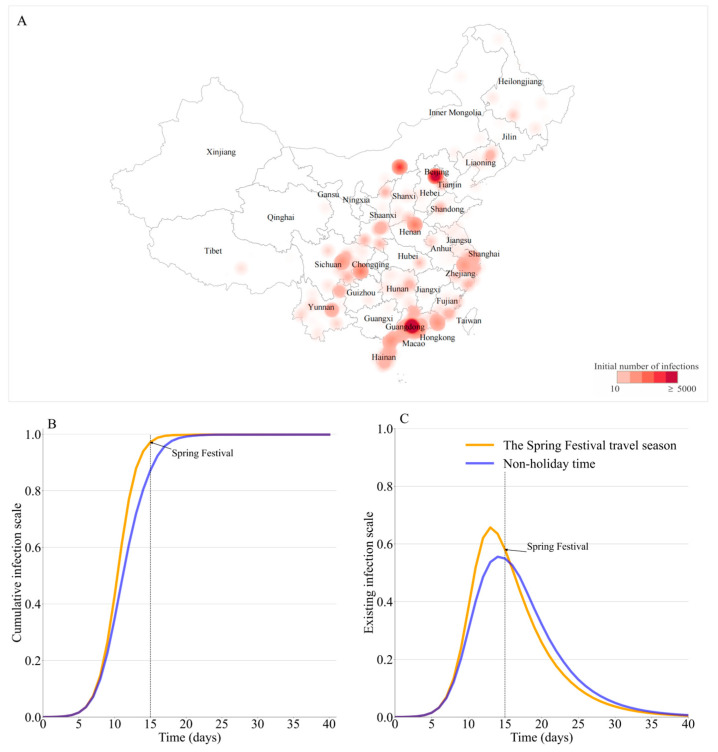
(**A**) The distribution of the initial seed of infection nationwide (darker colors indicate a larger initial infection size). (**B**) Cumulative infection scale and (**C**) existing infection scale over time for Spring Festival travel season and non-holiday time nationwide.

Under the initial condition of *R*_0_ = 15 and using the reported COVID-19 infection on 17 December 2022 as the initial condition (Figure 2A), both the non-holiday time and the Spring Festival travel season experience a rapid outbreak infecting almost the entire population. As shown in Figure 2B,C, for the non-holiday time and Spring Festival travel season, the peak timings are on day 14 and day 13 after model simulation with peak magnitudes of 0.56 and 0.66, respectively. The extremely high peak magnitudes are caused by the compound factors of a highly transmissible disease, an almost naive population, widespread initial infections, and intense population movement across cities.

**Figure 3:**
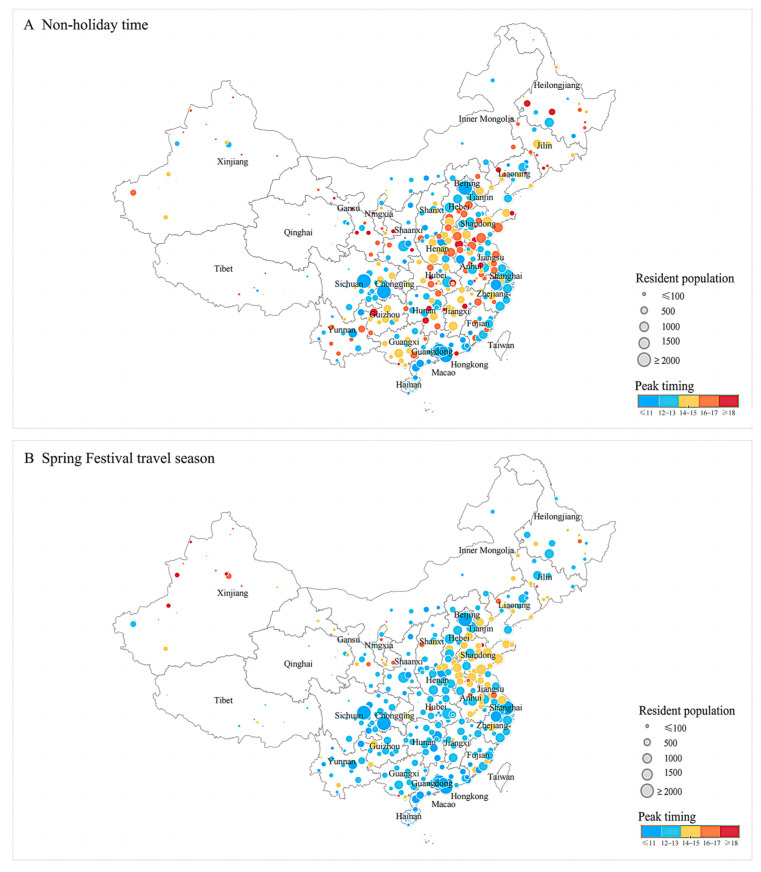
Peak timing in each city during (**A**) non-holiday time and (**B**) Spring Festival travel season. The unit of resident population is 10,000 people. The unit of peak timing is day. Peak timing is calculated as days after the first day in model simulation.

Cities with initial infections peak more quickly (before day 14) during the non-holiday time. During Spring Festival travel, most cities reach their peaks 1–4 days earlier compared to the non-holiday time and most cities without initial infections peak before day 14 as well (Figure 3).

**Figure 4:**
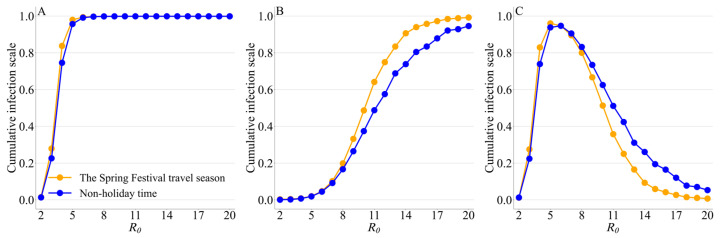
Under the conditions of different *R*_0_ and using the reported infection on 17 December 2022 as the initial seeds, the cumulative infection scale of (**A**) Spring Festival travel season, (**B**) pre-Spring Festival, and (**C**) post-Spring Festival. The blue line is the result of the same number of days of non-holiday time.

**Figure 5:**
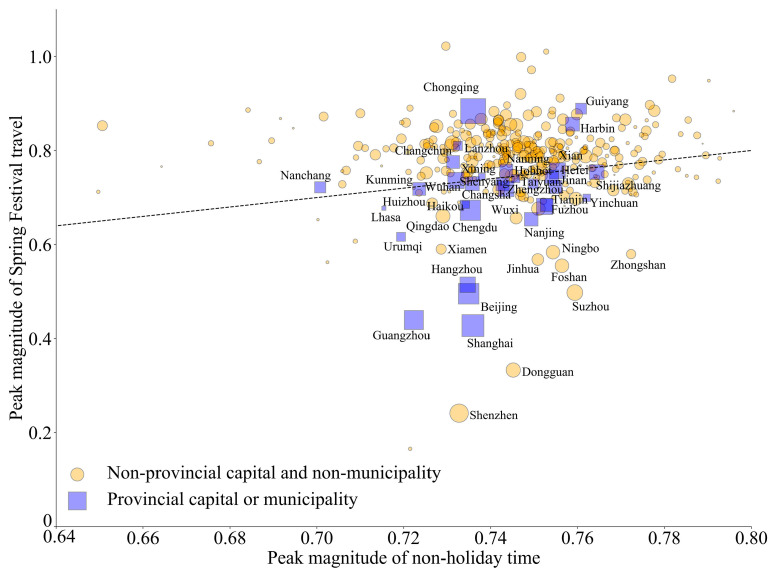
Peak magnitude in each city during non-holiday time and Spring Festival travel season. Peak magnitude is calculated as the fraction of active infections among residential population at peak timing. The size of the node indicates the number of residential populations. Blue squares are provincial capitals or municipalities. The dotted line is the diagonal line y = x. Note that peak magnitude can be greater than 1 because the number of infections in the city can be larger than residential populations due to influx from other cities.

As shown in Figure 4 when *R*_0_ = 15, most infections occur before the Spring Festival. Pre-Spring Festival travel brings a large number of infections from large cities to smaller cities. In addition, the migration from metropolitan areas greatly increases the susceptible pool in other cities. This results in a significantly higher peak magnitude in small- or medium-sized cities compared to the non-holiday time. In contrast, some large cities, such as Beijing, Shanghai, Guangzhou, Shenzhen, and Hangzhou, have lower peak magnitudes in the Spring Festival travel (Figure 5).

To more comprehensively analyze the impact of Spring Festival travel on the spread of different viruses, we set different *R*_0_ and used the reported infection on 17 December 2022 as the initial seeds for the simulation, where we mainly analyzed the impact of Spring Festival travel on the whole country and each city.

**Figure 6:**
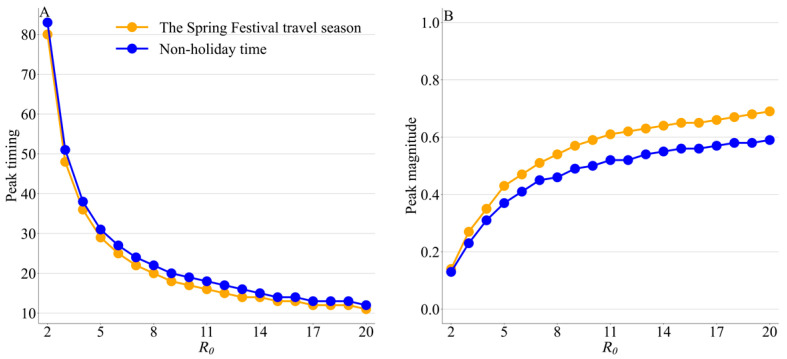
Under the conditions of different *R*_0_, (**A**) peak timing and (**B**) peak magnitude of the whole country during the non-holiday time and the Spring Festival travel season. The unit of peak timing is the day.

The large *R*_0_ and the unmitigated transmission are the main reasons the pathogen can spread rapidly in both scenarios. For other *R*_0_ values, Spring Festival travel can still accelerate peak timing at the national level, with a higher national peak magnitude compared to the non-holiday time. The increase in *R*_0_ can accelerate the national peak during the Spring Festival travel season and non-holiday time, with increased peak magnitudes. Further increases in *R*_0_ can reduce the difference in peak timing between the two periods from 3 days to 1 day, while the impact of increased *R*_0_ on the increased peak magnitude during Spring Festival travel is more pronounced for $R_{0}\;<\;10$ (Figure 6).

We further discussed the results for each city at *R*_0_ of 10, 5, and 2. Experimental results are shown in the Supplementary Materials. The increase in *R*_0_ allows the cities to reach the peak time faster and with a larger peak magnitude. Spring Festival travel can accelerate the peak timing for cities and make the non-initial infection cities peak on similar days as the initial infection cities. The peaks of most cities are reached before day 19, before day 31, and before day 84, respectively (Figures S1, S3 and S5). The acceleration of the peak timing by the Spring Festival travel is approximated under different *R*_0_. Therefore, we should pay more attention to the peak timing under each *R*_0_, analyze the epidemiological characteristics of each type of virus, assess its infectiousness, and thus make different preventive responses.

The four different peak timing and the growth in the cumulative infection scale around the Spring Festival (Figure 4) illustrate the epidemic spread of viruses with $R_{0}$ of 15, 10, 5, and 2 occurring mainly before the Spring Festival, around the Spring Festival, after the Spring Festival, and after the Spring Festival travel, respectively. As shown in Figure S2, the epidemic spread with $R_{0}$ of 10 is similar to with $R_{0}$ of 15, and influenced by mass flow from large cities to smaller cities, which also shows a decrease in the peak magnitude in large cities and an increase in the peak magnitude in small or medium cities during the Spring Festival travel.

The epidemic spread with $R_{0}$ of 5 is influenced by the large outflow from large cities before the Spring Festival and the return of people to large cities after the Spring Festival. During the Spring Festival travel, compared to non-holiday time, the reduction in the number of susceptible people within cities due to population outflow can result in lower peak magnitudes in some large cities, while population return can result in approximate or greater peak magnitudes in some large cities (Figure S4).

When *R*_0_ = 2, the population drifts between large and small cities, and mass population movements during the Spring Festival travel have less impact on the epidemic as the increase in cases mainly occurs after the Spring Festival travel, when the peak magnitude increases in some large cities, while there is no clear pattern in smaller cities (Figure S6). 

The temporal difference in the number of infections among travelers can dynamically impact the spatial distribution of infected and susceptible populations in all cities, which further determines the variation in local peak timing and magnitude. For instance, for a less transmissible disease (*R*_0_ = 5), peaks in large cities are delayed until after the Spring Festival, when a large number of susceptible and infected people return to large cities and fuel the epidemic spread. The peak magnitude is similar to or even higher than in the non-holiday time, which could stress the capacity of healthcare systems.

In summary, different *R*_0_ can have different effects on virus transmission, such as the difference in infection scale around the Spring Festival and the peak timing of infection nationwide and in each city. Therefore, in the process of epidemic prevention and control, preventive measures should be formulated according to the transmission characteristics of the virus, and medical resources should be dispatched based on the scale and peak timing of infection in each period. During the Spring Festival travel season, the asymmetry of population movement before and after the Spring Festival is a key factor to consider when assessing the risk of virus transmission. If the virus spreads mainly before the Spring Festival, the mobility characteristics of the population shifting to smaller cities make it appropriate to focus on infections in smaller cities; if the virus spreads mainly after the Spring Festival, we should focus on the importation of cases to large cities and the growth of infections within large cities.

## 4. Discussion

Travel during the Spring Festival holiday in China is one of the largest population migrations on earth. Using historic inter-city traffic flow data and a city-level epidemic transmission model, we simulated the epidemic spread across 371 Chinese cities under pre-pandemic travel conditions. We analyzed the impact of the massive population movement during the Spring Festival Travel on the spread of epidemics, focusing on peak timing and peak magnitude.

Using the epidemiological features of Omicron as an example, we performed extensive model simulations. At the national level, infection peaks before the Spring Festival and its timing are not particularly affected by the Spring Festival travel for a highly transmissible disease. However, peak magnitude for active infections increases by 0.1 compared to the non-holiday time.

At the city level, infectious people migrate to cities with few initial infections during Spring Festival travel, making their infection peak earlier than during the non-holiday time with considerably higher peak magnitudes. Our simulations imply that, after the relaxation of epidemic control policies, small- or medium-sized cities, usually with limited healthcare resources, may be more impacted by the epidemic, especially for highly transmissible viruses. Therefore, those cities with limited medical reserves and in rural areas should focus on adopting social distancing to flatten the epidemic curve, stocking medicines to reduce morbidity, and planning resources (ICUs, equipment, staffing) for patients with severe illness. For less transmissible pathogens, we should pay attention not only to the epidemic situation in small- or medium-sized cities during the homecoming period but also to the rapid epidemic spread in large cities during the returning period after holidays.

The impact of the Spring Festival travel on epidemic spread is compounded by several factors. First, population migration shapes the spatial distribution of infectious and susceptible populations across cities. The asymmetric traffic before and after the Spring Festival creates a temporal fluctuation of population present in each city, which strongly impact local transmission dynamics. Second, disease transmissibility determines the speed at which infections unfold within a population. The infection prevalence among travelers in the homecoming and returning traffic depends on the temporal growth of epidemics in each city. It is the combination of local infection dynamics and the timing of migration that collectively determines the effect on peak timing and magnitude.

There are several limitations in this modeling study, both in terms of data and model. The initial conditions of epidemic simulation (e.g., initial transmission time, initial infection size, and initial transmission seed) have a large impact on virus transmission, and specific analyses should be conducted for each type of situation, a single model with some parameters cannot profile all results. Tencent data do not fully cover mobility across all pairs of cities, which leads to the absence of some inter-city traffic flows. The model only considers the spatial variation of travelers on a particular day but does not consider the duration of the specific trip. This study does not consider other holidays covered during the simulation and mainly focuses on the differences between the Spring Festival travel and the non-holiday time. In reality, vaccination or mask-wearing and other measures will change model parameters, which will lead to different outcomes between the simulation results and in reality. This study does not take into consideration the effects of intrinsic fluctuations and the steady states of the model. We do not pay much attention to the dynamic behavior of the epidemic model.

## 5. Conclusions

While reducing epidemic prevention and control measures and restoring the normal life of the nation, research on various types of viruses should be maintained to reduce the harm to the economy and life from outbreaks caused by new viruses and virus mutations, and to focus on the differences between special periods and daily life to measure their impact on the epidemic to develop targeted measures.

The Spring Festival is an important traditional holiday in China, and the increase in overall national population travel during the Spring Festival travel can accelerate the epidemic spread and increase the peak magnitude of infections nationwide. Different infectious viruses affect the infection rate of the population returning home before the Spring Festival and the population returning to work after the Spring Festival, and cities have asymmetries in the inflow and outflow of population before and after the Spring Festival; these affect the peak time and peak magnitude of cities, with particular attention to the impact of highly infectious viruses with *R*_0_ > 10 on small- or medium-sized cities and the impact of viruses with *R*_0_ < 5 on large cities. This study provides a quantitative reference for Spring Festival travel epidemic prevention and control for regional governments in China.

## Data Availability

The data underlying this article will be shared on reasonable request to the corresponding author.

## Supplementary Materials

The following are available online at https://www.mdpi.com/article/10.3390/v15071527/s1, Figure S1: When *R*_0_ = 10, the peak timing of cities in (A) non-holiday time and (B) Spring Festival travel season. Figure S2: When *R*_0_ = 10, the peak magnitude in each city during non-holiday time and Spring Festival travel season. Figure S3: When *R*_0_ = 5, the peak timing of cities in (A) non-holiday time and (B) Spring Festival travel season. Figure S4: When *R*_0_ = 5, the peak magnitude in each city during non-holiday time and Spring Festival travel season. Figure S5: When *R*_0_ = 2, the peak timing of cities in (A) non-holiday time and (B) Spring Festival travel season. Figure S6: When *R*_0_ = 2, the peak magnitude in each city during non-holiday time and Spring Festival travel season.
