# Supplementary material for: The Impact of Spring Festival Travel on Epidemic Spreading in China
Source: Viruses. 2023 Jul 10;15(7):1527. doi: 10.3390/v15071527 (PMC10384880; doi:10.3390/v15071527)
Supplement: Supplementary file 1 [file viruses-15-01527-s001.zip › viruses-2434349-supplementary.pdf]

Supplementary Material

We further simulated the epidemic spread under different R_0 in supplementary information. Figure S1, S2, S3, S4, S5, S6 show the peak timing and peak magnitude for each city at R_0 of 10, 5, and 2.

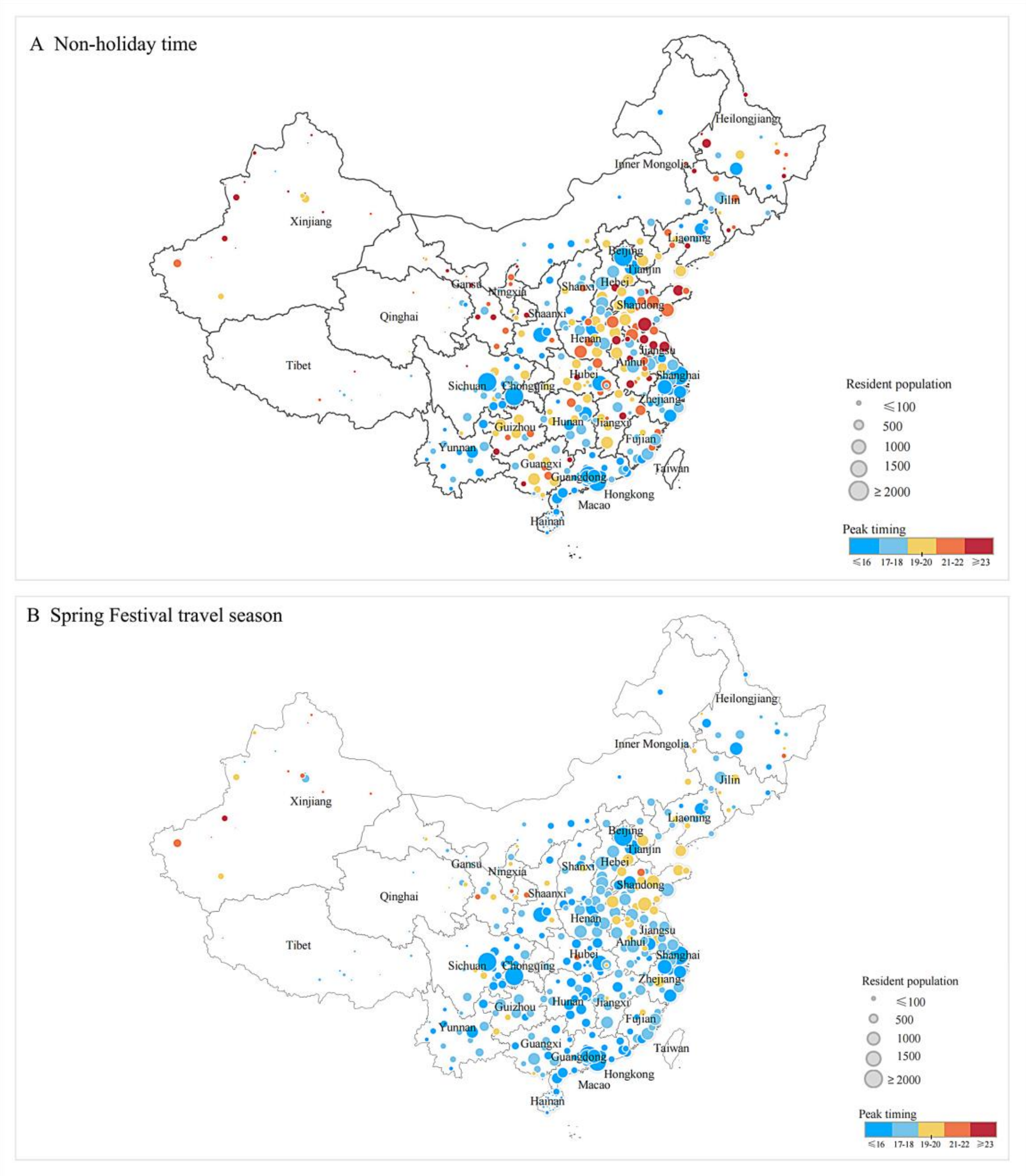

Figure S1 When R_0 = 10, the peak timing of cities in (A) non-holiday time and (B) Spring Festival travel season. The unit of resident population is 10,000 people. The unit of peak infection period is the day.

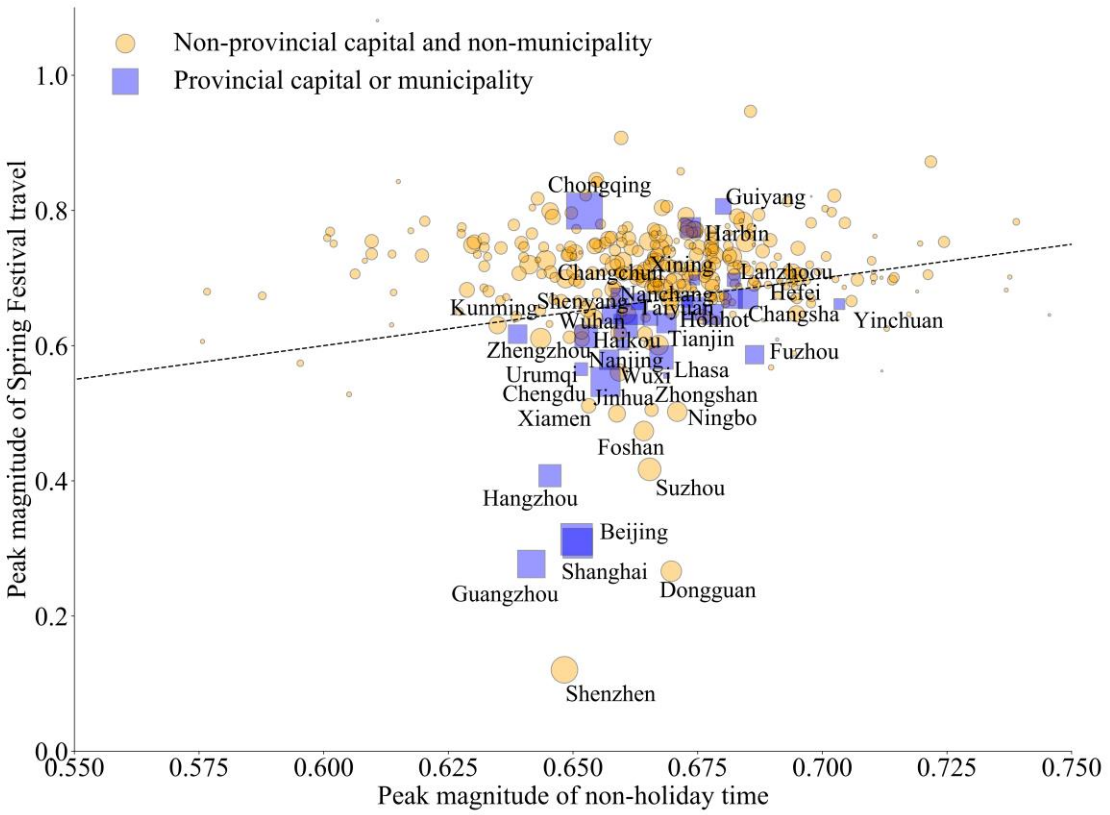

Figure S2 When R_0 = 10, the peak magnitude in each city during non-holiday time and Spring Festival travel season. The size of the node indicates the number of resident populations, and the blue square is the provincial capital or municipality. The dotted line is the diagonal line y=x.

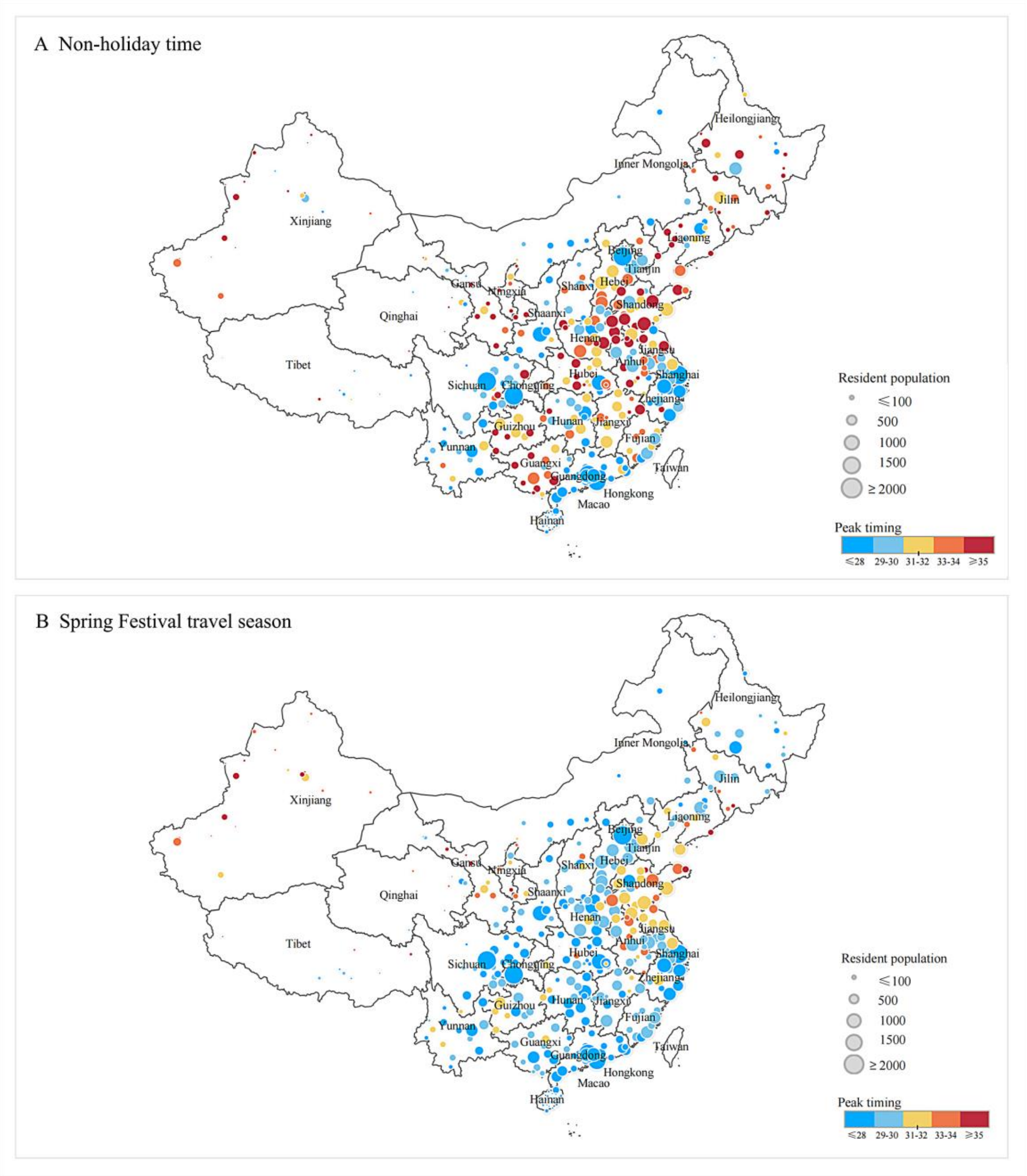

Figure S3 When R_0 = 5, the peak timing of cities in (A) non-holiday time and (B) Spring Festival travel season. The unit of resident population is 10,000 people. The unit of peak infection period is the day.

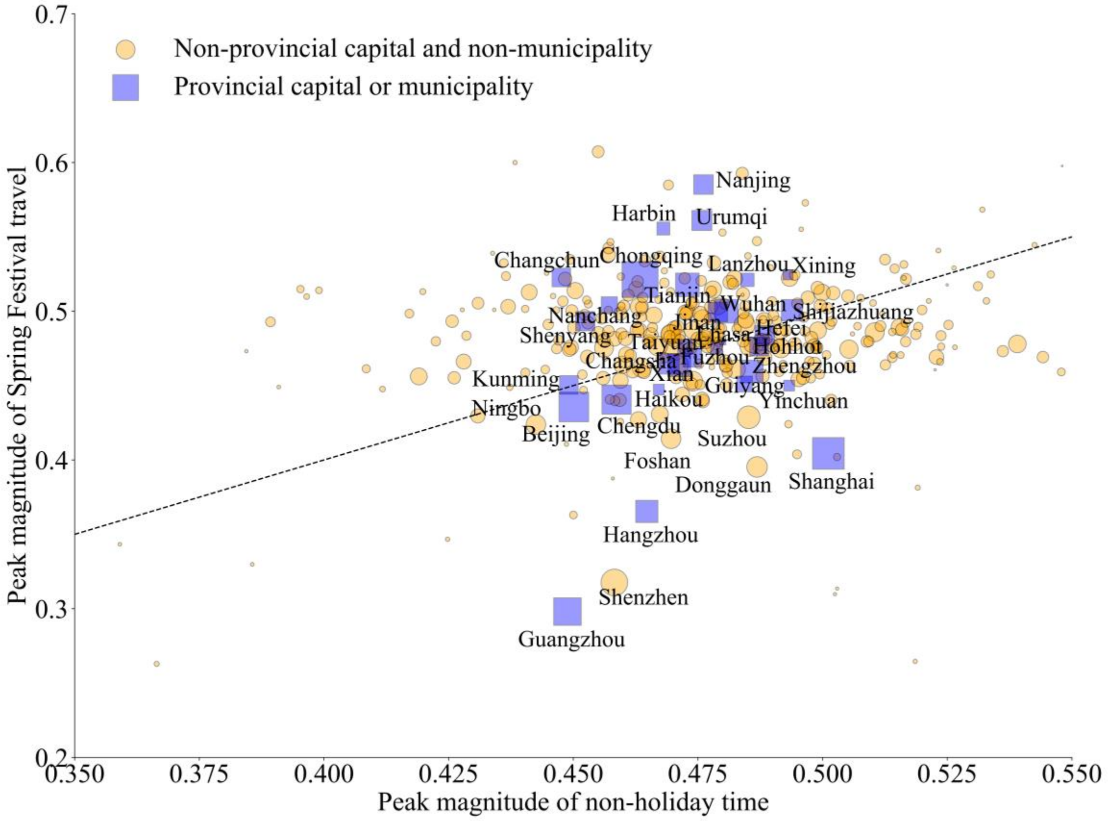

Figure S4 When R_0 = 5, the peak magnitude in each city during non-holiday time and Spring Festival travel season. The size of the node indicates the number of resident populations, and the blue square is the provincial capital or municipality. The dotted line is the diagonal line y=x.

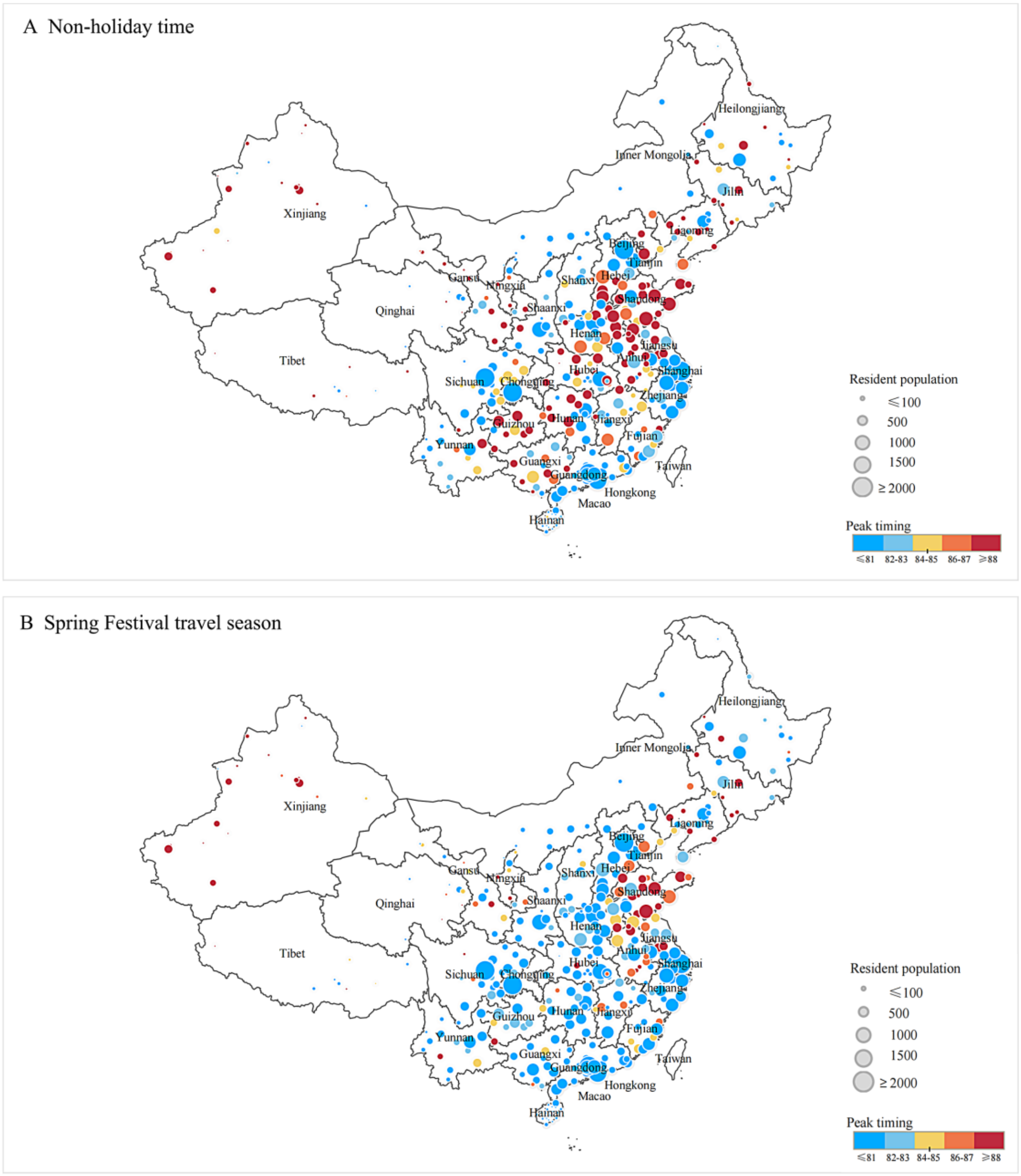

Figure S5 When R_0 = 2, the peak timing of cities in (A) non-holiday time and (B) Spring Festival travel season. The unit of resident population is 10,000 people. The unit of peak infection period is the day.

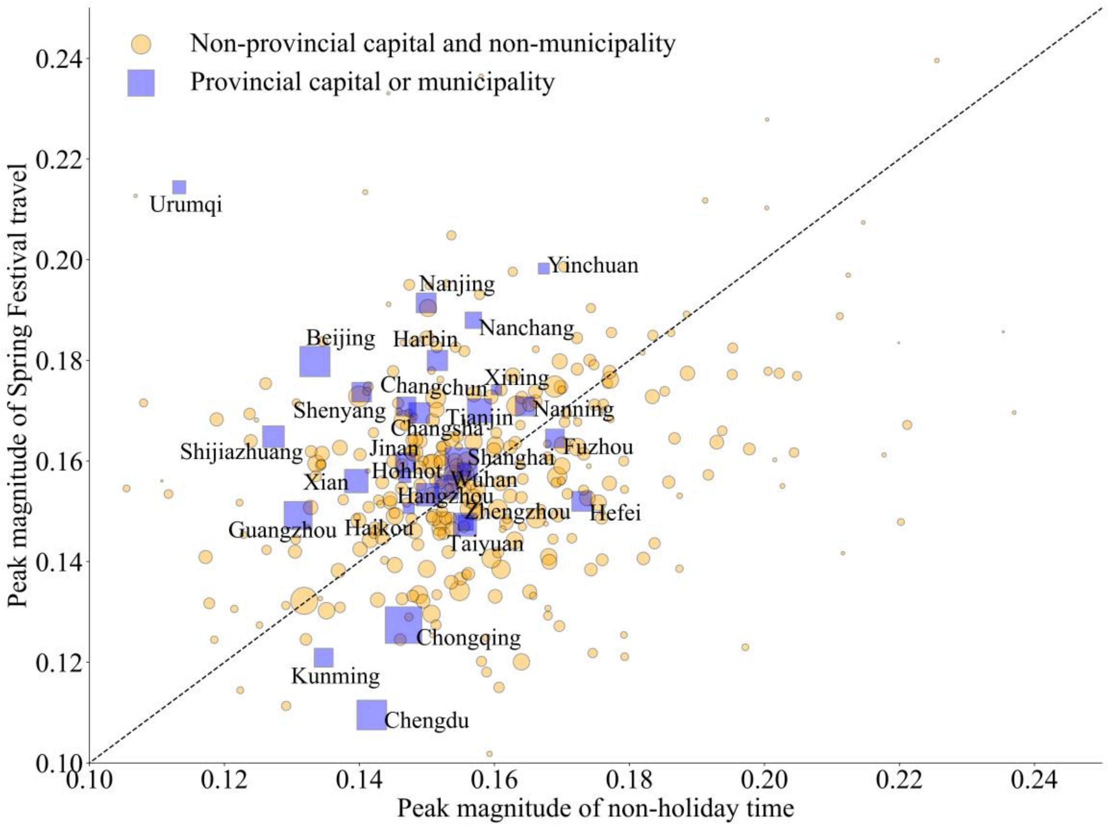

Figure S6 When R_0 = 2, the peak magnitude in each city during non-holiday time and Spring Festival travel season. The size of the node indicates the number of resident populations, and the blue square is the provincial capital or municipality. The dotted line is the diagonal line y=x.
